# Case Report: Prosthetic valve migration unmasking behçet disease after transcatheter aortic valve replacement

**DOI:** 10.3389/fcvm.2026.1947155

**Published:** 2026-10-28

**Authors:** Xi Wang, Yupei Qin, Wenjuan Bai

**Affiliations:** Department of Cardiology, West China Hospital, Sichuan University, Chengdu, China

**Keywords:** aortic regurgitation, behçet disease, case report, prosthesis migration, transcatheter aortic valve replacement

## Abstract

**Introduction:**

Behçet disease (BD) is a systemic vasculitis with protean manifestations; concurrent skeletal and cardiovascular involvement is exceptionally rare and poses formidable diagnostic challenges.

**Patient presentation:**

We report a 51-year-old woman who presented with multifocal osteolytic bone lesions and acneiform eruptions, initially fulfilling the Benhamou criteria for SAPHO syndrome. Severe aortic regurgitation was discovered, and transapical transcatheter aortic valve replacement (TAVR) was performed using the J-valve system to avoid sternotomy in the context of suspected active osteitis. At 3 months post-TAVR, the patient developed cerebral infarction and superior mesenteric artery thrombosis despite therapeutic anticoagulation. At 5 months, the prosthesis catastrophically dislodged into the left ventricular outflow tract. This event, occurring without evidence of infective endocarditis or procedural failure, prompted diagnostic reconsideration. The constellation of recurrent oral ulcers, acneiform lesions, multifocal osteolytic lesions, arterial thrombosis, and prosthetic valve dehiscence led to a revised diagnosis of BD-associated aortitis. Histopathological examination of the explanted tissue revealed lymphocytic infiltration and focal necrosis without infection. Following a 3-month course of immunosuppression, the patient underwent a Bentall procedure and remained asymptomatic with sustained cardiac reverse remodeling at 2-year follow-up.

**Conclusion:**

Prosthetic valve dehiscence in the absence of infection should be regarded as a sentinel diagnostic event for inflammatory aortitis, even when formal BD classification criteria are initially unmet. This case underscores the imperative of establishing a unifying diagnosis before committing to structural cardiac intervention in patients with concurrent systemic inflammatory features.

## Introduction

Behçet disease (BD) is a systemic vasculitis of uncertain etiology capable of affecting vessels of all sizes, with a clinical spectrum ranging from mucocutaneous lesions to life-threatening vascular complications (1, 2). Cardiac involvement — most commonly aortic regurgitation secondary to aortitis or valvulitis — occurs in a minority of patients (3). Destructive skeletal involvement is rarer still, described only in isolated case reports (4–7). The concurrent presentation of severe osteoarticular and cardiovascular disease therefore represents an exceptional diagnostic challenge.

SAPHO syndrome (Synovitis, Acne, Pustulosis, Hyperostosis, and Osteitis) is a distinct clinical entity characterized by sterile osteoarticular inflammation and characteristic dermatological findings, most commonly palmoplantar pustulosis and severe acne (8). Critically, SAPHO syndrome carries no reported cardiovascular involvement — a feature that, in principle, should serve as a key discriminator when cardiac pathology coexists with osteo-cutaneous disease.

Transcatheter aortic valve replacement (TAVR) is primarily indicated for elderly patients with aortic valve disease or those at elevated surgical risk; it may also be considered in younger patients when conventional sternotomy carries specific procedural hazards (9). In the present case, the clinical diagnosis of SAPHO syndrome prompted selection of TAVR specifically to avoid sternotomy and the attendant risk of impaired bone healing in the setting of suspected active osteitis — a decision that would ultimately prove fateful.

Herein, we report a case in which catastrophic TAVR prosthesis migration ultimately unmasked underlying BD-associated aortitis. This case illustrates the diagnostic significance of prosthetic valve dehiscence as a sentinel event for inflammatory aortitis and highlights the imperative of establishing a unifying diagnosis before committing to structural cardiac intervention in patients with concurrent systemic inflammatory features.

## Case description

A 51-year-old woman presented with a 2-year history of multifocal bone pain and acute inability to ambulate due to severe lower back pain (Table 1). Prior imaging at an external facility had revealed multiple osteolytic lesions, and a clavicular biopsy showed bone marrow edema, which was reported only as suggestive of chronic osteomyelitis without definitive evidence of infection. She had a positive T-SPOT.TB assay and a strongly positive purified protein derivative skin test. In the absence of a definitive diagnosis, she received empiric anti-tuberculosis therapy; however, her symptoms progressively worsened after 1 month, prompting transfer to our institution.

**Table 1:** Timeline of the clinical episode of care.

Time Point	Event
2 years prior to admission	Onset of multifocal bone pain; no definitive diagnosis
1 month prior to admission	Empiric anti-tuberculosis therapy at external facility; symptoms worsened
Admission (Day 0)	Transfer to our institution. Multifocal osteolytic lesions, acneiform eruptions, recurrent oral ulcers, dyspnea (NYHA II–III), diastolic murmur, severe aortic regurgitation on TTE. SAPHO syndrome diagnosed per Benhamou criteria; diclofenac started (pain score 7→2)
1 month post-discharge	Readmission for worsening heart failure. Pre-TAVR CT: tricuspid AV, annulus 23.8 mm, sinus 31.3 mm, LVOT 27.3 mm, no root calcification
TAVR procedure	Transapical J-valve 25-mm self-expanding prosthesis deployed successfully; TEE confirms no paravalvular leak. Discharged Day 5 on warfarin
3 months post-TAVR	Dizziness, limb weakness. Cerebral MRI: right occipital ischemic lesion. CT angiography: superior mesenteric artery thrombus. Second-degree type II atrioventricular block detected; dual-chamber pacemaker implanted. INR 1.9. TTE: normal prosthesis function, no paravalvular leak. Hospitalized, symptoms resolved
5 months post-TAVR	Chest tightness, fatigue. TTE+CT: prosthesis migration into LVOT with severe paravalvular regurgitation. No infective endocarditis. Diagnostic revision: BD-associated aortitis
5–8 months post-TAVR	3-month immunosuppressive bridging (prednisone, thalidomide, cyclophosphamide); inflammatory markers significantly reduced
8 months post-TAVR	Bentall procedure with St. Jude Medical 23-mm mechanical valved conduit. Intraoperative finding: friable, inflamed aortic annulus. Histopathology: lymphocytic infiltration, focal necrosis, no infection, no IgG4 + plasma cells
Surgery+2 years	Asymptomatic, no bone pain recurrence. TTE: LVEF 58%→67%, LVEDD 60→48 mm, normal mechanical prosthesis function
Latest follow-up	Sustained clinical and echocardiographic remission

AV, aortic valve; CT, computed tomography; INR, international normalized ratio; LVEDD, left ventricular end-diastolic dimension; LVEF, left ventricular ejection fraction; LVOT, left ventricular outflow tract; MRI, magnetic resonance imaging; NYHA, New York Heart Association; TEE, transesophageal echocardiography; TTE, transthoracic echocardiography.

At evaluation, the patient reported dyspnea on mild exertion [New York Heart Association (NYHA) functional class II–III] and recurrent oral ulcers. Physical examination revealed a blood pressure of 130/70 mm Hg, a heart rate of 80 beats per minute, multiple acneiform lesions on the face, a diastolic murmur at the aortic valve area, percussion tenderness over the lumbar spine and ribs, and mild bilateral lower extremity edema. She had an 8-year history of essential hypertension managed with long-term medication.

Given the progressive deterioration despite one month of empirical anti-tuberculosis therapy — a course that argued against active tuberculosis — the diagnosis was reconsidered. The differential included connective tissue diseases, metabolic bone disorders, bone malignancies, autoimmune liver disease, thalassemia, and systemic vasculitides. Laboratory investigations revealed significant microcytic anemia (hemoglobin, 80 g/L) and a profound systemic inflammatory response (erythrocyte sedimentation rate >120 mm/h, C-reactive protein 77 mg/L, serum amyloid A 149 mg/L, and alpha-1 acid glycoprotein 1,920 mg/L). Serum and urine immunofixation electrophoresis were negative for monoclonal protein, and serum free light chain analysis demonstrated a normal kappa-to-lambda ratio, effectively excluding multiple myeloma. Serum calcium and parathyroid hormone levels were within normal limits. Comprehensive autoimmune profiling was unremarkable, excluding major systemic autoimmune rheumatic diseases and autoimmune hepatitis. Serum immunoglobulin G subclass analysis demonstrated an IgG4 level of 1.42 g/L. HLA-B27 and HLA-B51 typing were negative, and a pathergy test was negative. At this stage, the patient's International Criteria for Behçet Disease (ICBD) score was 3 points — recurrent oral aphthosis (2 points) and acneiform skin lesions (1 point) — falling below the 4-point diagnostic threshold, and BD was therefore considered unlikely given the additional absence of genital ulcers, uveitis, or a positive pathergy test (3). IgG4-related disease was not extensively pursued given the lack of characteristic multi-organ involvement and normal serum IgG4 level, although IgG4 immunohistochemistry was not performed on the clavicular biopsy specimen because the patient declined further testing.

Computed tomography (CT) of the chest and spine revealed hyperostosis and sclerosis involving the right sternoclavicular and first sternocostal joints, accompanied by joint surface irregularities and subchondral cystic changes. Multifocal inflammatory involvement extended to the cervical (C6–7), thoracic, and lumbar spine (L2–5), manifesting as facet joint erosion and partial vertebral fusion (Figure 1A, Supplementary Video S1). Whole-body bone scintigraphy confirmed multifocal radiotracer uptake in the sternum, right sternoclavicular joint, and multiple vertebrae (Figures 1B,C). Although multifocal spinal lesions can mimic seronegative spondyloarthropathies, ankylosing spondylitis was excluded by negative HLA-B27 typing and the absence of sacroiliitis or syndesmophytes. The convergence of prominent anterior chest wall involvement, multifocal osteolytic lesions, and cutaneous acne fulfilled the diagnostic criteria proposed by Benhamou et al., establishing a clinical diagnosis of SAPHO syndrome (10).

**Figure 1:**
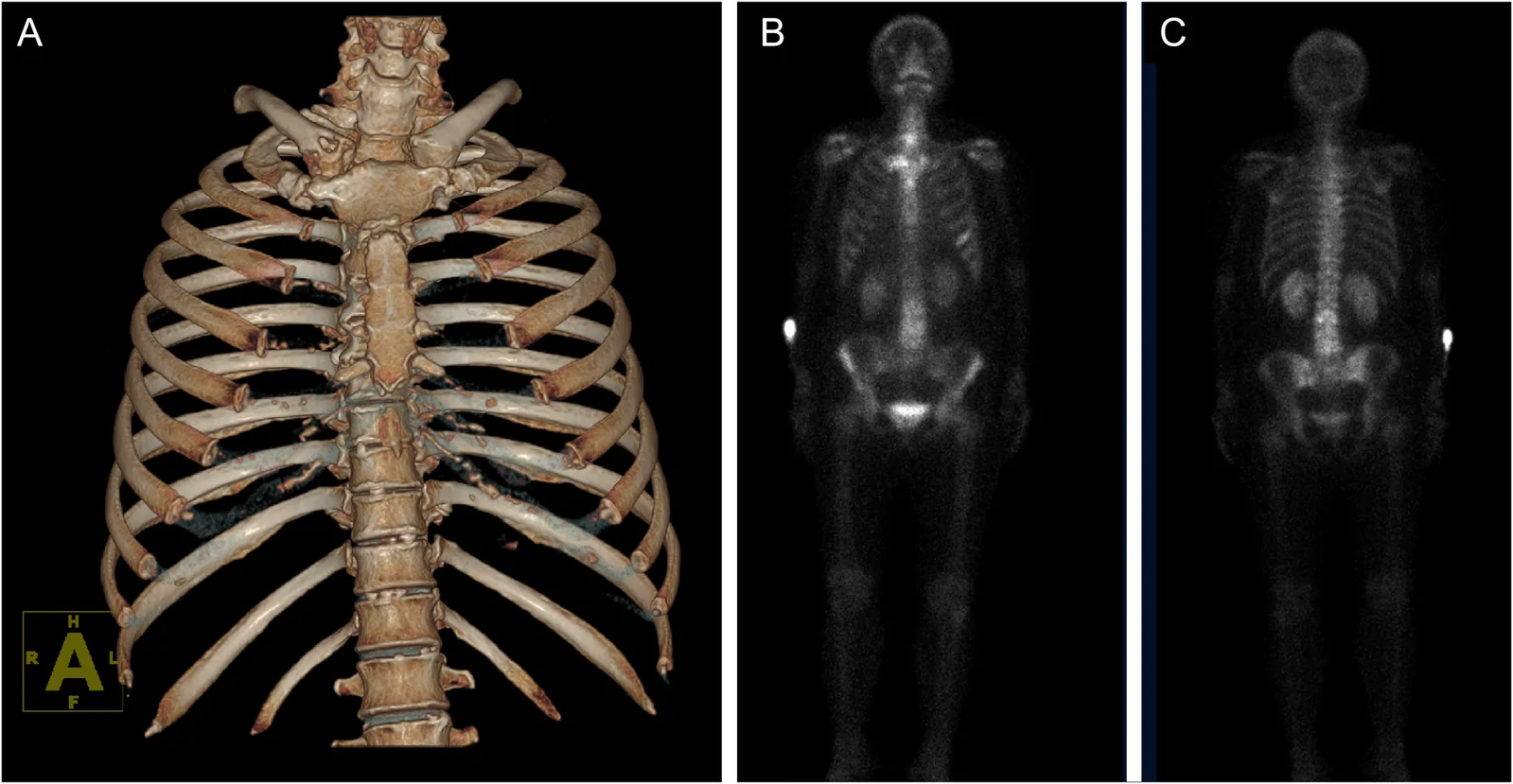
Multimodality imaging of osteoarticular lesions. **(A)** Three-dimensional reconstructed chest CT revealing multiple bony erosions with a “moth-eaten” appearance and surrounding sclerosis involving the sternoclavicular joint and spine. **(B,C)** Whole-body bone scintigraphy confirming multifocal radiotracer uptake in the sternum, right sternoclavicular joint, and multiple vertebrae.

Transthoracic echocardiography (TTE), prompted by the patient’s heart failure symptoms, revealed severe aortic regurgitation (vena contracta, 7 mm) with left ventricular dilation (end-diastolic dimension, 60 mm) and preserved systolic function (ejection fraction, 58%) (Figure 2A).

**Figure 2:**
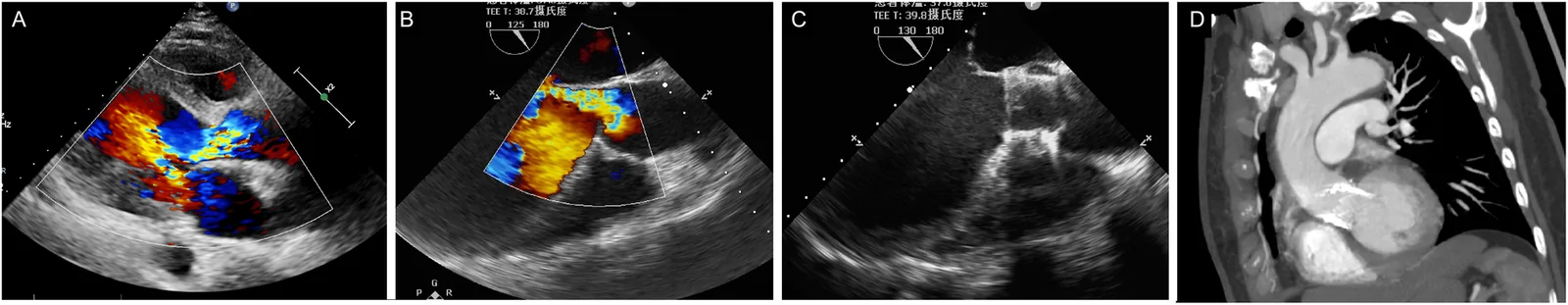
Multimodality imaging before and after transcatheter aortic valve replacement. **(A)** Preoperative transthoracic echocardiography demonstrating severe aortic regurgitation with left ventricular dilation. **(B)** Intraoperative transesophageal echocardiography (TEE) pre-deployment demonstrating severe aortic regurgitation (eccentric jet). **(C)** Post-deployment TEE confirming stable prosthesis position with tight apposition to surrounding tissues and no paravalvular leak.**(D)** CT at 3-month follow-up demonstrating stable prosthetic valve positioning.

The patient was started on diclofenac, which reduced her pain score from 7 to 2, and was discharged. One month later, she presented with worsening heart failure and was readmitted. The decision to pursue TAVR over surgical aortic valve replacement was driven by concerns that sternotomy might impair bone healing in the context of suspected SAPHO-related active osteitis. Pre-TAVR CT angiography revealed a tricuspid aortic valve with an annular diameter of 23.8 mm, an average sinus diameter of 31.3 mm, and a left ventricular outflow tract (LVOT) diameter of 27.3 mm, without root calcification — confirming anatomical suitability for transcatheter valve implantation (Supplementary Video S2).

The transapical TAVR procedure was performed using the J-valve system (Genesis MedTech, China), a self-expanding transcatheter heart valve designed for treating aortic regurgitation in the absence of annular calcification (11). The J-valve device incorporates three U-shaped nitinol graspers deployed centrally within the three aortic sinuses to achieve prosthesis fixation and protect the coronary ostia from obstruction. The procedure was performed under general anesthesia with fluoroscopic guidance and continuous transesophageal echocardiography (TEE) monitoring (Figure 2B). Transapical access was achieved via a small intercostal incision over the left ventricular apex. The three U-shaped nitinol graspers were sequentially placed into the aortic sinuses in the standard 3-cusp coplanar fluoroscopic view. A 25-mm self-expanding valve was then deployed and securely seated within the graspers. Intraoperative TEE and aortic root angiography confirmed optimal valve position with normal leaflet function, no paravalvular leak, and stable prosthesis anchorage (Figure 2C; Supplementary Video S3). The patient was discharged 5 days later on warfarin for thromboprophylaxis.

At the 3-month follow-up, the patient presented with dizziness and limb weakness. Cerebral magnetic resonance imaging revealed a small acute ischemic lesion in the right occipital lobe, and CT angiography identified a thrombus in the superior mesenteric artery. TTE confirmed normal prosthetic valve function with a peak transvalvular velocity of 1.6 m/s and no paravalvular leak, and CT imaging demonstrated stable valve positioning (Figure 2D). The international normalized ratio was 1.9. Electrocardiography revealed second-degree type II atrioventricular block (Mobitz II), for which a dual-chamber permanent pacemaker was implanted. She was hospitalized for observation and discharged after complete symptom resolution.

At 5 months post-TAVR, the patient presented with chest tightness and fatigue. TTE demonstrated partial migration of the prosthetic valve into the left ventricular outflow tract, resulting in severe paravalvular regurgitation spanning from 11 to 5 o'clock. Notably, the prosthesis itself remained structurally intact and functionally normal, with normal leaflet opening and a peak forward flow velocity of 2.6 m/s (Figure 3A; Supplementary Video S4, S5). CT confirmed distal migration of the prosthetic valve approximately 10 mm below the native annular plane into the left ventricular outflow tract, with the proximal portion remaining anchored within the aortic root, together with aortic wall thickening consistent with aortitis (Figure 3B). This catastrophic complication, occurring without evidence of infective endocarditis (repeatedly negative blood cultures, absence of vegetations) or procedural failure, coupled with the antecedent arterial thrombotic events and multifocal bone destruction, prompted a diagnostic re-evaluation. The constellation of recurrent oral ulcers, acneiform skin lesions, osteolytic bone lesions, arterial thrombosis, and prosthetic valve dehiscence strongly suggested BD-associated vasculitis as the unifying etiology.

**Figure 3:**
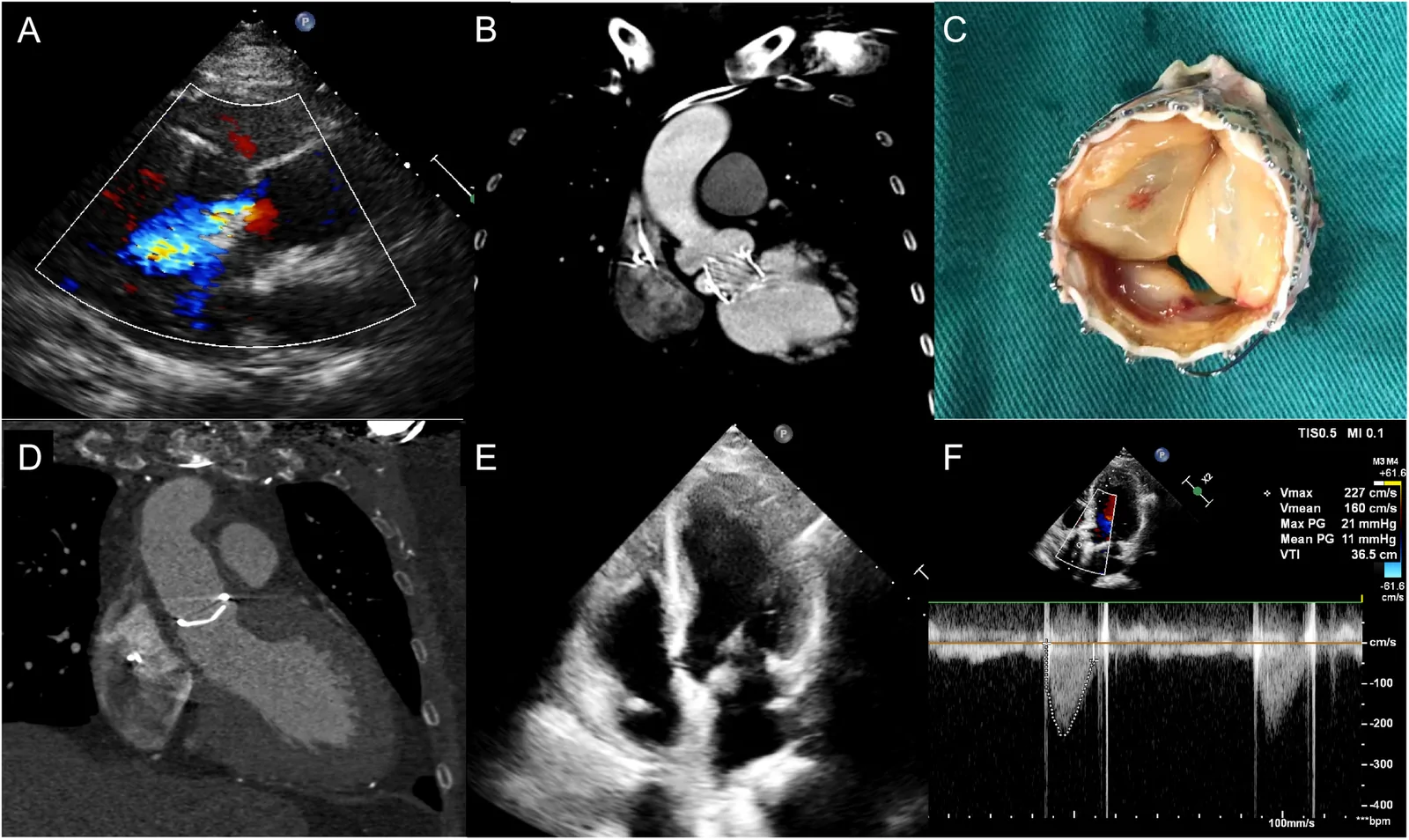
Imaging of prosthesis migration and revision surgery. **(A)** Color Doppler transthoracic echocardiography demonstrating severe paravalvular regurgitation due to prosthesis migration. **(B)** CT demonstrating distal migration of the prosthetic valve approximately 10 mm below the native annular plane into the left ventricular outflow tract, together with aortic wall thickening consistent with aortitis. **(C)** Photograph of the explanted 25-mm J-valve prosthesis following surgical retrieval. **(D)** CT following Bentall procedure showing the surgically implanted mechanical valve prosthesis and ascending aortic graft in appropriate position. **(E)** Apical four-chamber view at the 2-year follow-up demonstrating sustained reverse remodeling. **(F)** Continuous-wave Doppler in the apical three-chamber view at the 2-year follow-up demonstrating normal mechanical prosthesis function with a peak forward flow velocity of 2.3 m/s and a mean gradient of 11 mmHg.

To mitigate the risk of suture dehiscence associated with active vasculitis, the patient first underwent a 3-month course of combination immunotherapy (prednisone, thalidomide, and cyclophosphamide), which significantly reduced inflammatory markers. Following immunosuppressive bridging, she underwent open-heart surgery for explantation of the migrated transcatheter prosthesis and a Bentall procedure using a St. Jude Medical 23-mm valved conduit (Figures 3C,D). Intraoperatively, the native aortic annulus appeared friable and inflamed, consistent with active sterile aortitis rather than infective endocarditis; the explanted prosthesis was macroscopically intact with no vegetations or other signs of infection. Histopathological examination of the excised aortic root tissue revealed uneven valvular thickening, focal myxoid degeneration, lymphocytic infiltration, and focal necrosis on the intimal surface, without evidence of infective organisms, granulomatous inflammation, storiform fibrosis, or IgG4-positive plasma cell infiltration.

The patient tolerated the procedure well and was discharged on warfarin. Over the following 2 years, she remained asymptomatic with no recurrence of bone pain and no tuberculous reactivation, despite substantial prior immunosuppressive exposure. Serial TTE demonstrated sustained reverse remodeling: left ventricular ejection fraction improved from 58% to 67%, and left ventricular end-diastolic dimension decreased from 60 mm to 48 mm (Figure 3E), and continuous-wave Doppler confirmed normal mechanical valve function with a peak forward flow velocity of 2.3 m/s and a mean gradient of 11 mmHg at the latest follow-up (Figure 3F).

## Discussion

This case presents a diagnostically challenging intersection of two rare disease phenotypes — BD-associated osteoarticular destruction and aortitis — whose convergence initially satisfied criteria for SAPHO syndrome, an entirely different disease with no reported cardiovascular involvement. The diagnostic error was perpetuated because the severe aortic regurgitation was evaluated as an incidental finding rather than as the cardiovascular manifestation of the same systemic process. To our knowledge, this is the first reported case of late TAVR prosthesis migration attributed to BD-associated aortitis.

A central challenge was that, at initial evaluation, the patient did not satisfy the International Study Group (ISG) 1990 criteria (2) and scored only 3 points on the International Criteria for Behçet Disease (ICBD) 2014 (3) — recurrent oral aphthosis (2 points) and acneiform skin lesions (1 point) — falling below the 4-point diagnostic threshold; BD was therefore dismissed. The subsequent cerebral infarction and superior mesenteric artery thrombosis added a point for vascular involvement, bringing the ICBD score to 4 and thereby meeting the diagnostic threshold. Yet these features were not synthesized into a unifying diagnosis until the catastrophic prosthesis migration at 5 months. This diagnostic gap, in which cardiovascular events precede the recognition of BD, is in fact the norm rather than the exception in BD-related cardiac disease. This delayed recognition reflects, in part, the unreliability of conventional exclusion criteria in such phenotypes — notably, the absence of HLA-B51 does not exclude BD, and cardiovascular involvement may constitute the initial recognized manifestation in some patients (12). Han et al. (12) reported that only 2 of 15 patients (13.3%) with surgically confirmed BD-related aortic regurgitation had been diagnosed with BD before their first cardiac operation; the remaining 13 were identified only after developing characteristic postoperative complications, most commonly prosthetic valve dehiscence. In the largest surgical series to date, Choi et al. (13) found that only 17.1% of 35 patients met ISG criteria preoperatively, and approximately 25% remained undiagnosed by either ISG or ICBD criteria even at surgery. The 2-year incidence of paravalvular leak in BD patients was 51.4%, compared with approximately 1% in non-BD controls (13). Patients with incomplete criteria BD had paradoxically worse outcomes precisely because their BD went unrecognized, delaying postoperative immunosuppressive therapy (13).

In our patient, the prosthetic valve migration — occurring without evidence of infective endocarditis, with therapeutic anticoagulation, and without identifiable procedural shortcomings — was the pivotal clinical event. While the existing surgical literature attributes valve failure to aortitis-induced annular friability causing suture dehiscence (13, 14), our case highlights a distinct mechanism specific to transcatheter valves: inflammation-mediated compromise of the tissue integrity required for the J-valve's three U-shaped nitinol graspers to maintain secure apposition, a process that may have contributed to the displacement of the entire stent-valve complex. Unlike balloon-expandable valves, which derive their anchoring from radial force against a calcified annulus, the J-valve depends critically on the integrity of the aortic sinus wall — rendering it uniquely vulnerable in the setting of active aortitis. Histopathology of the excised aortic tissue showed lymphocytic infiltration and focal necrosis without evidence of infection. Although these findings are not specific for Behçet aortitis, they supported a sterile inflammatory aetiology and, together with the absence of granulomatous inflammation and IgG4-positive plasma cells, argued against the principal alternative diagnoses (infective endocarditis and IgG4-related disease). Although the 2014 ICBD criteria incorporated vascular manifestations into the scoring system — and our patient ultimately met the diagnostic threshold once these arterial events accrued (3) — this case reinforces the notion that severe prosthetic valve complications in the absence of infection should be regarded as a sentinel diagnostic event for inflammatory aortitis, even when the significance of the individual features is not recognized in real time.

This case further highlights the diagnostic complexity of BD's skeletal manifestations. Although articular involvement in BD is well recognized, destructive osteolytic lesions are decidedly uncommon. Several case reports have described BD-related erosive bone lesions affecting a wide range of joints (4–7). In our patient, involvement of both the spine and sternoclavicular joints closely mimicked the characteristic anatomical pattern of SAPHO syndrome — a resemblance that, combined with the presence of acne, was sufficient to satisfy the diagnostic criteria proposed by Benhamou et al. (10) and obscure the true diagnosis. Notably, the osteolytic lesions had been present for approximately two years before any corticosteroid exposure — corticosteroids were initiated only after the diagnosis of BD was made — excluding a steroid-induced etiology and indicating that the bone destruction was intrinsic to the underlying disease process.

Regarding the decision to proceed with TAVR in this patient, we acknowledge that current guidelines favor surgical aortic valve replacement for a 51-year-old patient. The initial rationale — avoiding sternotomy to preserve bone healing in the context of suspected SAPHO-related active osteitis — was based on an incomplete diagnostic framework at the time. In retrospect, performing TAVR without first controlling the underlying inflammatory process placed a rigid prosthetic device into an actively inflamed aortic annulus, an environment in which the J-valve's graspers, critically dependent on sinus wall tissue integrity for anchorage, were fundamentally compromised. As demonstrated by Choi et al. (13), preoperative immunosuppression alone does not prevent paravalvular leak; rather, aortic root replacement combined with postoperative immunosuppression is required. The optimal pathway would have been early recognition of a unifying inflammatory etiology, induction of remission through immunosuppression, and definitive Bentall procedure.

In conclusion, when unexplained aortic regurgitation coexists with systemic inflammatory features, a unifying diagnosis must be pursued and disease control established before committing to structural cardiac intervention. Prosthetic valve dehiscence in the absence of infection should raise strong suspicion for underlying inflammatory aortitis and trigger diagnostic reconsideration, even when formal classification criteria are initially unmet.

## Patient perspective

The patient provided written informed consent for the publication of this case report. After experiencing a protracted diagnostic journey marked by two misdiagnoses, progressive symptom deterioration, and a catastrophic procedural complication, the patient expressed relief at finally receiving an accurate diagnosis and definitive surgical treatment. She reported satisfaction with her current quality of life.

## Data Availability

The datasets presented in this article are not readily available because the patient data primarily consists of medical imaging images, which contain personally identifiable information. Requests to access the datasets should be directed to the corresponding author.

## Supplementary material

SUPPLEMENTARY VIDEO S1 Three-dimensional reconstructed chest CT revealing multiple bony erosions.

SUPPLEMENTARY VIDEO S2 Pre-TAVR CT angiography demonstrating aortic root anatomy.

SUPPLEMENTARY VIDEO S3 Intraoperative TEE confirming stable prosthesis deployment without paravalvular leak.

SUPPLEMENTARY VIDEO S4 TTE at 5 months demonstrating migration of the prosthetic valve into the left ventricular outflow tract.

SUPPLEMENTARY VIDEO S5 Color Doppler flow imaging demonstrating severe paravalvular regurgitation.
